# The burden of neck pain in Brazil: estimates from the global burden of disease study 2019

**DOI:** 10.1186/s12891-021-04675-x

**Published:** 2021-09-21

**Authors:** Lucas de Melo Castro Deligne, Maria Clara Brant Rocha, Deborah Carvalho Malta, Mohsen Naghavi, Valéria Maria de Azeredo Passos

**Affiliations:** 1 Faculdade Ciências Médicas de Minas Gerais, Belo Horizonte, Minas Gerais Brazil; 2 School of Nursing, Universidade Federal de Minas Gerais, Belo Horizonte, Minas Gerais Brazil; 3 Institute of Health Metrics and Evaluation, Washington University, Seattle, USA; 4 School of Medicine, Universidade Federal de Minas Gerais, Belo Horizonte, Minas Gerais Brazil

**Keywords:** Neck pain, Burden of disease, Prevalence, Years lived with disability, Disability-adjusted life years

## Abstract

**Background:**

This study analyzed neck pain estimates in Brazil and its states between 2000 and 2019, in view of the country’s lacking epidemiological data.

**Methods:**

An analysis was performed of the GBD 2019 estimates by location, sex, and age, per 100,000 population, with uncertainty intervals (95% UI). Brazilian estimates were compared to global, Mexican, English, and American rates.

**Results:**

Global, Brazilian, and Mexican prevalence numbers were statistically homogeneous and stable in the period. Throughout the period analyzed in the study, Brazilian neck pain prevalence (2241.9; 95%UI 1770.5–2870.6) did not show statistical differences when compared to global (2696.5; 95%UI 2177.0–3375.2) or Mexican (1595.9; 95%UI 1258.9–2058.8) estimates. Estimates observed in the USA (5123.29; 95%UI 4268.35–6170.35) and England (4612.5; 95%UI 3668.8–5830.3) were significantly higher. In 2019, when compared to the USA and England, age-standardized prevalences were lower globally, in Brazil, and in Mexico. Prevalences in Brazilian states were similar, being that Roraima (1915.9; 95%UI 1506.5–2443.1) and the Federal District (1932.05; 95%UI 1515.1–2462.7) presented the lowest and highest values respectively. The exception was the state of São Paulo (3326.5; 95%UI 2609.6–4275.5). There was no statistical difference by sex, but the prevalence tended to increase with aging. In 2019, the Brazilian prevalence was 2478.6 (95% UI 1791.0–3503.8), 5017.2 (95%UI 3257.26–7483.8), and 4293.4 (95% UI 2898,8–6343.9), for those aged 15 to 49, 50 to 69, and 70+ years. There was no statistical difference among the YLDs in all locations and times.

**Conclusions:**

Brazil is going through a fast-paced process of populational aging; a higher prevalence of neck pain in middle-aged individuals and the elderly highlights the need for lifelong prevention initiatives. The higher rates observed among higher-income populations and the homogeneity of the Brazilian estimates suggest a lack of robust epidemiological data in lower-income countries.

## Introduction

Musculoskeletal diseases are the main causes of disability among adults [1, 2]. Much of this disability burden is associated with populational aging, but also with behavioural and work-related risk factors [3]. Among musculoskeletal diseases, neck pain is the second most common cause of disability, second only to low back pain [4].

From a global perspective, neck pain has a point prevalence of 14.4%, while the mean lifetime prevalence is 23.1% [5]. The 12-month prevalence estimates vary from 21 to 42% among children and adolescents to 30 to 50% among adults and the elderly [6]. In Brazil, in 2017, this prevalence among adults was estimated at 20.3% [7].

Neck pain is a complex condition, in which the association of individual, ergonomic, socio-cultural and psychosocial risk factors contributes to its occurrence and chronicity [8]. The main individual factors are age, sex, increased body mass index and smoking [9, 10]. Among the environmental factors are those related to ergonomics, such as strenuous physical activity, use of force and vibration, improper posture and repetitive movements [6]. Previous history of neck and low back pain, poor health care and psychosocial problems such as job dissatisfaction, stress, anxiety and depression are other factors related to neck pain [10].

The economic impact of neck pain is significant and includes costs related to treatment, decreased productivity, non-attendance to work and social security [11]. In the USA, neck pain was responsible for 16 million medical consultations in 2010 [12]; in 2016, health costs for neck and low back pain diagnosis and treatment were estimated at 134 billion dollars [13]. In 2012, neck pain was responsible for job absences of 25.5 million Americans, who missed an average of 11.4 days of work [1]. Approximately three out of five European workers experienced symptoms of musculoskeletal pain in 2015, with neck and upper limbs pain accounting for 41% of the complaints [14]. In Brazil, neck pain was responsible for 7.2% of disability pensions granted to workers with musculoskeletal diseases [15].

The prevalence and the disability burden of neck pain in Brazil, both countrywide and at state level, have not yet been well defined, despite its likely impact on the economy and health of the Brazilian population. Therefore, this study aims to describe the prevalence and the burden of neck pain in Brazil and in the units of the federation in the years 2000, 2010 and 2019, as well as to draw parallels with the estimates of other countries for the same years.

## Methodology

This descriptive study is based on the estimates from the Global Burden of Diseases Study (GBD) 2019, a systematic effort in descriptive epidemiology coordinated by the Institute for Health Metrics and Evaluation (IHME) at the University of Washington [16]. All estimates come from interdisciplinary approaches to enhance data quality and statistical modeling that have been already reported [16]. Since 2014, several investigations on the burden of disease were conducted by the GBD Brazil Network, which was created as a collaboration among the IHME, the Brazilian Ministry of Health, and a network of academic institutions led by the Federal University of Minas Gerais [17].

Neck pain is defined as pain located between the occipital region and the third thoracic vertebra, with or without irradiation to one or both upper limbs, which lasts more than 1 day and limits daily activities [18, 19]. The definition of neck pain was based on the codification by the International Classification of Diseases, tenth edition (ICD-10): neck pain (M54.2); cervicocranial syndrome (M53.0); cervicobrachial syndrome (M53.1); back pain with cervical radiculopathy (M54.1); cervical spondylosis with radiculopathy (M47.2); cervical spondylosis with myelopathy (M47.1); cervical disc disorder (M50); cervicogenic headache (R51); sprain of ligaments of cervical spine (S13.4); and sprain of joints and ligaments of other parts of neck (S13.8) [20].

The GBD study uses three main indicators to calculate the burden of diseases: years of life lost due to premature mortality (YLLs), years lived with disability (YLDs) and the sum of YLLs to YLDs, which corresponds to the disability-adjusted life years (DALYs) [21]. Since neck pain is not a cause of death but a disabling condition, YLD and DALY estimates for neck pain are the same.

The disability weights used for estimating the diseases’ YLDs are measured on a scale of zero to one, in which zero is equivalent to full health and one is equivalent to death. Disability weights were obtained from surveys conducted in several countries from different regions. The surveys used paired-comparison questions, in which the respondents considered two hypothetical individuals with different, randomly selected health conditions and were asked to indicate which individual they considered to be the healthiest. More methodological details are available elsewhere [22].

YLD due to neck pain is determined by multiplying the specific disease disability weight (dw) by its prevalence in a given population (p) [23]:

$$ \mathrm{YLD}=\kern0.5em \mathrm{DW}\kern0.5em \mathrm{x}\kern0.5em \mathrm{P},\mathrm{where}\ {\mathrm{YLD}}_{\mathrm{NP}}\kern0.5em =\kern0.5em \left(0.04\kern0.5em \mathrm{x}\ \mathrm{P}\mathrm{revalence}\ \mathrm{of}\ \mathrm{mild}\ \mathrm{acute}\ \mathrm{sequelae}\right)\kern0.5em +\kern0.5em \left(0.221\kern0.5em \mathrm{x}\kern0.5em \mathrm{P}\mathrm{revalence}\kern0.5em \mathrm{of}\kern0.5em \mathrm{severe}\ \mathrm{acute}\ \mathrm{sequelae}\right)\kern0.5em +\kern0.5em \left(0.101\kern0.5em \mathrm{x}\kern0.5em \mathrm{P}\mathrm{revalence}\ \mathrm{of}\ \mathrm{mild}\ \mathrm{chronic}\ \mathrm{sequelae}\right)\kern0.5em +\kern0.5em \left(0.286\kern0.5em \mathrm{x}\kern0.5em \mathrm{P}\mathrm{revalence}\ \mathrm{of}\ \mathrm{severe}\ \mathrm{chronic}\ \mathrm{sequelae}\right) $$

The Socio-Demographic Index (SDI) is an indicator directly correlated to social economic standards used to compare different locations and times. It consists of a scale between zero and one, which aggregates the per capita income, educational level for those ages 15 and older, and fertility rate of women under 25 years of age. The higher the SDI, the greater the location’s socio-demographic development is [18].

Neck pain prevalence and YLD estimates were analyzed for Brazil and all of its federative units. Global estimates and estimates for other countries were also used to compare the magnitude of national burden: 1) Mexico, a country in Latin America with an SDI similar to that of Brazil; 2) United States of America (USA), a country with one of the highest prevalence and YLD rates for neck pain; and 3) England, a developed country with a public health system similar to Brazil’s. For each location, data were generated by both sexes, by standardized age, or by age group in the years 2000, 2010 and 2019.

Estimates were standardized by age to allow for a comparative analysis that takes into account changes due to population growth and aging. In addition, the position and changes in the classification rank of neck pain prevalence and YLDs were analyzed in the selected locations, in the same years of 2000, 2010 and 2019. To describe the degree of confidence in the metrics, the modeling process enables estimates to be calculated 1000 times and then distributed from the lowest to the highest value. The 95% uncertainty interval (95% UI) is obtained from the 2.5 and 97.5 percentiles of this distribution, and it considers the error generated by the sample, the modeling, and the availability of data. All estimates are presented with their 95% UI, and differences between estimates are statistically significant if the intervals are not coincident [16]. All estimates were produced by the Institute for Health Metrics and Evaluation (IHME) and available in the IHME website at: http://ghdx.healthdata.org/gbd-results-tool [7]. The authors are responsible for describe and analyze these data.

The input data for neck pain in Brazil was based on three studies [7, 15, 24], covering the period from 2011 to 2017, two of them conducted in the state of São Paulo [7, 24]. The data sources are available in the Global Health Data Exchange for each country (http://ghdx.healthdata.org/countries). Neck pain prevalence was estimated using the software DisMod-MR 2.1, by Bayesian meta-regression modeling tool [16]. The full list of articles used for statistical modelling can be found in the GBD 2019 Data Input Sources Tool [20]. To estimate prevalence, incidence, and YLDs, the Bayesian meta-regression tool DisMod-MR 2.1 is used [16]. The modeling allows obtaining the best estimates, mainly of the localities such as Brazil, with few surveys and studies. In the GBD study, data are estimated in a homogeneous way, making it possible to compare neck pain between locations, by sex, and by age group.

The GBD Brazil study uses only secondary data, and it does not identify the individual or requires a free and informed consent form. The study was approved by the Ethics Committee of the Federal University of Minas Gerais (UFMG) under the registration n° 628,033,167.00005149, according to the resolution no. 466/2012 of the National Health Council of Brazil.

## Results

In Brazil, the age-standardized prevalence of neck pain in 2019 was 2241.9 (95% IU 1770.6 – 2870.6) per 100,000 population. This means that in that year, about five million of the 211 million Brazilians presented with neck pain. Also in 2019, the global age-standardized prevalence was 2696.5 (95% IU 2177 – 3357.2) per 100,000 population, similar to the Brazilian estimates. Throughout the study period, neck pain prevalence in Brazil did not show statistical differences when compared to global or Mexican estimates. On the other hand, the prevalence estimates of the USA and England were, in general, significantly higher (Table 1).

**Table 1:** Prevalence and YLD estimates from 2000 to 2019. Estimates from the GBD 2019

Prevalence and YLD per 100,000 population (95%UI)						
Both Sexes, Age-Standardized^a^						
		2000	2010	2019	▲v%^b^ 2000–2010	▲v% 2010–2019
Global SDI^c^ (0.56–0.65)	Prevalence	2698.4 (2167.3-3386.2)	2614.5 (2120.3-3276.1)	2696.5 (2177-3375.2)	−3.11	3.14
	YLD	267.7 (175.9–384.2)	259.7 (170.4–370)	267.4 (175.5–383.5)	−2.99	2.93
Brazil SDI (0.54–0.64)	Prevalence	2252.3 (1779.1-2883.3)	2625 (2077.2-3322.1)	2241.9 (1770.6-2870.6)	16.55	−14.59
	YLD	221.9 (145.80–322)	259.1 (170.1–373.1)	221.7 (145.4–322.6)	16.77	−14.43
Mexico SDI (0.56–0.65)	Prevalence	1595 (1258.5-2057.9)	1595.6 (1258.7-2058.6)	1595.9 (1258.9-2058.9)	0.04	0.018
	YLD	158.2 (104.9–230.6)	158 (104.5–229.7)	157.8 (104.5–229.2)	−0.13	− 0.07
United States SDI (0.85–0.91)	Prevalence	3984.8 (3195.3-5057)	2718.6 (2386.3-3116.7)	5123.3 (4268.4-6170.4)	−31.78	88.46
	YLD	391 (257.3–558.9)	264.8 (182.4–362.9)	500.3 (338.9–704.9)	−32.28	88.94
England SDI (0.79–0.85)	Prevalence	5255.4 (4218.8-6665)	3816.5 (3069.3-4750.6)	4612.6 (3668.8-5830.3)	− 27.38	20.86
	YLD	520.7 (344.2–754)	377.9 (250.1–543.1)	457.7 (309–651)	− 27.41	21.10

Source: IHME, GBD Study 2019, available at http://ghdx.healthdata.org/gbd-results-tool. ^a^95% UI: 95% uncertainty intervals, ^b^▲v%-percentage change, ^c^SDI: Social-Demographic Index

Most of the neck pain prevalence values remained stable in the locations and periods surveyed. Only in the USA there was a significant increase of the prevalence: 88.46% between 2010 and 2019, with the prevalence varying from 2718.6 (95% IU 2386.3 – 3116.7) cases per 100,000 population to 5123.3 (95% IU 4268.4 – 6170.4) cases per 100,000 population in the period. Prevalence rates in Brazil showed an inverse trend when compared to the other locations, increasing between 2000 and 2010 but decreasing from 2010 to 2019 (Table 1).

The prevalence in all Brazilian federative units remained stable. In the year 2000, these values varied from 1911.2 (95% IU 1503.3-2436.2) per 100,000 population in Roraima to 1932.2 (95% IU 1515.7-2462.3) per 100,000 population in Rio de Janeiro. In 2019, these values varied from 1915.9 (95% IU 1506.5-2443.1) per 100,000 population in the state of Roraima to 1932.1 (95% IU 1515.1-2462.7) per 100,000 population in Distrito Federal. The only exception occurred in the state of São Paulo, with prevalence values of 3325.7 (95% IU 2608.9 - 4274.5), 3698.6 (95% IU 3009.8 - 4592.2), and 3326.5 (95% IU 2609.7 – 4275.5) per 100,000 population in 2000, 2010, and 2019 respectively (Table 2).

**Table 2:** Prevalence and YLD estimates in Brazilian Federative Units from 2000 to 2019. Estimates from the GBD Study 2019

Prevalence and YLD per 100,000 population (95%UI)						
Brazil, Both Sexes, Age-Standardized						
		2000	2010	2019	▲v% 2000–2010	▲v% 2010–2019
**Northern States**						
Acre SDI (0.56)	Prevalence	1922.5 (1510.6-2450.9)	2295.2 (1781.8-2971.9)	1922.5 (1510.6-2450.9)	19.64	− 16.24
	YLD	189 (124.4–275.4)	226.1 (148.8–327)	189.8 (125.6–276.80)	19.68	−16.07
Amapá SDI (0.64)	Prevalence	1919.4 (1508.8-2445.5)	2294.4 (1781.9-2969.4)	1922.5 (1510.4-2451.1)	19.53	− 16.21
	YLD	189.7 (124–275.6)	227.1 (148.2–327.6)	190.3 (125.1–277)	19.76	−16.02
Amazonas SDI (0.60)	Prevalence	1919.6 (1509-2446.8)	2294.1 (1781.3-2968.4)	1921.2 (1509.9-2449.3)	19.51	−16.25
	YLD	190.1 (124.9–276.8)	227.5 (147.6–328.4)	190.6 (124.8–276.6)	19.70	−16.25
Pará SDI (0.57)	Prevalence	1919 (1508.9-2446)	2293.7 (1781-2968.2)	1921.3 (1509.9-2449.3)	19.52	−16.24
	YLD	189.4 (124.4–275.6)	226.9 (147.1–329.7)	190.4 (125.7–274.80)	19.80	−16.08
Rondônia SDI (0.60)	Prevalence	1911.5 (1502.7-2438.2)	2287.8 (1776-2962.1)	1919.3 (1508.8-2447.3)	19.68	−16.11
	YLD	188.5 (124.1–274.1)	226 (146.4–325.1)	189.7 (125.2–274.4)	19.91	−16.06
Roraima SDI (0.61)	Prevalence	1911.2 (1503.3-2436.2)	2286.7 (1774.9-2961)	1915.9 (1506.5-2443.1)	19.65	−16.21
	YLD	188.4 (124.4–274.3)	225.6 (145.2–326.1)	189.3 (125.8–276.1)	19.77	−16.07
Tocantins SDI (0.58)	Prevalence	1915.1 (1505.9-2442.1)	2289.6 (1778-2964.8)	1920.2 (1509.1-2448.7)	19.55	−16.13
	YLD	188.9 (124.8–275.7)	226.2 (146.9–326.5)	189.8 (123.8–274.8)	19.77	−16.08
**Northeastern States**						
Alagoas SDI (0.52)	Prevalence	1927.8 (1512.7-2458.4)	2307.8 (1793-2991)	1930.7 (1514.4-2462.3)	19.71	−16.34
	YLD	189.9 (125.7–275.8)	227.6 (148.7–330.8)	190.8 (125.8–276.4)	19.86	−16.20
Bahia SDI (0.56)	Prevalence	1926 (1512.1-2455.4)	2303.9 (1790-2983.3)	1928 (1513.4-2457.4)	19.62	−16.32
	YLD	189.8 (124.4–274.2)	227.4 (150–327.6)	190.5 (125.1–275.4)	19.76	−16.21
Ceará SDI (0.56)	Prevalence	1928.8 (1513.5-2459.4)	2306.7 (1792-2988.1)	1928.6 (1513.6-2458.5)	19.59	−16.39
	YLD	190.2 (125.7–275.6)	228.1 (149.1–330.4)	191 (124.8–276.3)	19.87	−16.23
Maranhão SDI (0.44)	Prevalence	1924.7 (1511.7-2454.1)	2300.4 (1786.2-2980.3)	1926.3 (1512.4-2455.7)	19.52	−16.26
	YLD	190.1 (125.6–278.6)	227.3 (147.7–326.2)	190.5 (124.5–277.7)	19.56	−16.17
Paraíba SDI (0.55)	Prevalence	1930.3 (1514.1-2461.2)	2308.6 (1793.9-2990.1)	1929.4 (1514.1-2459.1)	19.60	−16.42
	YLD	190.6 (126–275.6)	228.2 (148.4–330.9)	191 (126–277.3)	19.70	−16.28
Pernambuco SDI (0.57)	Prevalence	1931.4 (1514.8-2462.8)	2311.1 (1796-2994.3)	1931.5 (1515.2-2461.7)	19.66	−16.43
	YLD	190.4 (126.3–276.9)	228.4 (148.3–333.6)	190.8 (125.8–275.7)	19.94	−16.43
Piauí SDI (0.51)	Prevalence	1926.7 (1512.3-2457.1)	2304.5 (1789.9-2985.8)	1927.3 (1512.8-2457.3)	19.61	−16.37
	YLD	190.7 (124.4–279.5)	228.2 (148.5–331.1)	191.1 (125.7–278.2)	19.66	−16.23
Rio Grande do Norte SDI (0.58)	Prevalence	1928.5 (1513.3-2458.9)	2306.6 (1792.1-2987)	1928.2 (1513.5-2457.3)	19.6	−16.4
	YLD	190.5 (125.4–277.7)	228.1 (148.3–329.1)	190.9 (125.4–275.9)	19.77	−16.3
Sergipe SDI (0.58)	Prevalence	1928.4 (1513.1-2458.2)	2307.5 (1793-2989.3)	1930.1 (1514.4-2460.9)	19.66	−16.35
	YLD	190 (125.7–276.2)	227.9 (148.6–331.1)	190.9 (125.8–277)	19.99	−16.27
**Center-Western States**						
Goias SDI (0.63)	Prevalence	1921.8 (1509.8-2450.9)	2299.5 (1785.5-2978.5)	1924.6 (1511.6-2453.2)	19.65	−16.3
	YLD	189.3 (126–274.5)	226.9 (147.8–326.9)	190.3 (125–277.8)	19.84	−16.10
Distrito Federal SDI (0.78)	Prevalence	1931 (1515-2462.2)	2311.8 (1796.6-2996)	1932 (1515.1-2462.7)	19.72	−16.43
	YLD	190.1 (125.9–278.4)	228.3 (150–330.8)	191.3 (126–277.7)	20.06	−16.20
Mato Grosso SDI (0.64)	Prevalence	1913.2 (1503.7-2439.4)	2288.6 (1776.7-2962.5)	1918.8 (1508.7-2446.3)	19.63	−16.16
	YLD	188.7 (123.9–274.5)	226.2 (149.6–329.7)	189.9 (124.6–276.7)	19.89	−16.06
Mato Grosso do Sul SDI (0.64)	Prevalence	1920.8 (1509.5-2450.1)	2298.4 (1784.5-2977.1)	1923.8 (1511.2-2452.3)	19.66	−16.30
	YLD	189 (125–273.5)	226.2 (149.4–325.7)	189.9 (125.6–275.6)	19.69	−16.05
**Southeastern States**						
Espirito Santo SDI (0.66)	Prevalence	1924.7 (1511.4-2453.5)	2302.5 (1788.8-2981)	1926.1 (1512.7-2454.5)	19.63	−16.35
	YLD	189.8 (125.4–275.5)	227.5 (148.3–329.6)	190.6 (124.9–278.7)	19.90	−16.22
Minas Gerais SDI (0.64)	Prevalence	1925.7 (1512.2-2454.3)	2302.6 (1789-2981.1)	1925.8 (1512.4-2454.3)	19.58	−16.37
	YLD	190.1 (124.9–275.4)	227.7 (148.5–329.8)	190.8 (126.1–276.7)	19.80	−16.19
Rio de Janeiro SDI (0.70)	Prevalence	1932.2 (1515.7-2462.3)	2311.4 (1797-2993.4)	1930.8 (1514.9-2460.3)	19.63	−16.47
	YLD	191 (126.2–276.3)	228.9 (149.5–330.8)	191.4 (126.1–277.8)	19.84	−16.38
São Paulo SDI (0.70)	Prevalence	3325.7 (2608.9-4274.5)	3698.6 (3009.8-4592.2)	3326.5 (2609.7-4275.5)	11.21	−10.06
	YLD	326.6 (214.6–474.7)	364.1 (241.7–518.6)	328.5 (216.3–476.6)	11.45	−9.77
**Southern States**						
Paraná SDI (0.66)	Prevalence	1924.5 (1511.5-2453.9)	2303.2 (1789-2983.1)	1926.7 (1512.8-2455.7)	19.68	−16.34
	YLD	189.8 (124.7–274.2)	227.5 (148.6–330.1)	190.9 (126.3–278.3)	19.85	−16.09
Rio Grande do Sul SDI (0.68)	Prevalence	1928 (1513.6-2456.3)	2305.5 (1792.1-2984)	1927.1 (1513.3-2455.5)	19.58	−16.41
	YLD	189.9 (124.5–276.5)	227.8 (147.6–330.7)	190.6 (126.4–276)	19.92	−16.31
Santa Catarina SDI (0.69)	Prevalence	1924.6 (1511.9-2452.1)	2301.4 (1788.4-2978)	1924.6 (1512.-2451.9)	19.58	−16.37
	YLD	190.1 (125.1–275.5)	227.7 (149.3–328.7)	190.8 (125.7–277.9)	19.78	−16.24

Both sexes and age-standardized (A.S) from Brazilian federal units prevalences and YLDs rates in 2000, 2010 and 2019, and percentage change (▲v%) from 2000 to 2010 and from 2010 to 2019. Social-Demographic Index (SDI) from 2019

The annual change in neck pain prevalence estimates in Brazil was 16.55% between 2000 and 2010 and − 14.59% between 2010 and 2019. In the state of São Paulo, this variation was 11.21% between 2000 and 2010, and − 10.06% between 2010 and 2019. The average variation among Brazilian states was 20% between 2000 and 2010 and − 16% between 2010 and 2019. Although there is a difference between these variations, the magnitude of the curves for prevalence patterns in Brazil, the state of São Paulo, and the rest of the states was similar.

Table 3 shows 2019 global prevalence estimates of neck pain by sex and age groups, as well as age-standardized estimates for the countries evaluated. The prevalence is statistically higher for women in the USA, with the same trend occurring in the other countries. In Brazil, in 2019, the prevalence was 5376 (95% IU 3495.7-8035.3) per 100,000 population among women and 3611.2 (95% IU 2994.3- 6999.1) per 100,000 population among men.

**Table 3:** Prevalence of neck pain, by sex and age from 2000 to 2019. GBD 2019 estimates

Prevalence per 100,000 population (95%UI^a^)								
By sex and age range, age-standardized								
		Male				Female		
		2019	▲v%^b^ 2000–2010	▲v% 2010–2019		2019	▲v% 2000–2010	▲v% 2010–2019
Global SDI^c^ (0.56–0.65)	Age (Years)				Age (Years)			
	A.S	2352.3 (1907.3-2.951,9)	−2.86	3.02	A.S	3031.8 (2440.2-3.827,1)	−3.31	3.23
	15–49	2415 (1759-3353.4)	−3.01	3.51	15–49	3133.8 (2297.4-4345.1)	−1.9	4.12
	50–69	5210.8 (3485.7-7817.9)	−0.95	4.74	50–69	6885.3 (4599.1-10,306.2)	−1.63	4.63
	≥70	5344.1 (3600.5–7686.7)	0.08	4.53	> 70	6462.1 (4461.7-9238.7)	− 2.83	4.69
Brazil SDI (0.54–0.64)	Age (Years)				Age (Years)			
	A.S	2080.8 (1647.6-2657.6)	14.60	−13,16	A.S	2386.4 (1871.5-3060.2)	18.00	− 15.68
	15–49	2299 (1673.7-3254)	20.26	−8.07	15–49	2653.6 (1906.5-3770.1)	23.38	−10.13
	50–69	4611.2 (2994.3-6999.1)	17.86	−15.25	50–69	5376 (3495.7-8035.3)	22.60	−18.43
	≥70	3944.9 (2613.6-5820.3)	9.80	−8.93	> 70	4549 (3095.8-6719.4)	12.83	−12.19
Mexico SDI (0.56–0.65)	Age (Years)				Age (Years)			
	A.S	1496.9 (1183.4-1889.6)	0	0	A.S	1685.2 (1333.2-2166.3)	0	0
	15–49	1581 (1142.8-2212)	5.91	3.63	15–49	1815 (1317.3-2578.4)	7.27	4.34
	50–69	3234.1 (2123.2-4862.9)	−0.019	0.071	50–69	3645.5 (2405.4-5524)	−0.039	0.076
	≥70	2982 (1998.3-4376.5)	0.0095	0.15	> 70	3401.7 (2237.6-4990.3)	−0.43	−0.078
United States SDI (0.85–0.91)	Age (Years)				Age (Years)			
	A.S	4145.1 (3439.8-4972.2)	−21	−8.48	A.S	6077.7 (5039.6-7365.9)	−9	3.14
	15–49	4305.6 (3363.3-5590)	−19.75	− 9.25	15–49	7074.3 (5518-9244.4)	−6.04	1.53
	50–69	8853 (6306.5-12,529.6)	−22.69	−10.83	50–69	11,888.4 8394.4-16,535.7	− 10.17	6.22
	> 70	9502.4 (6954.3-12,866.8)	− 21.93	− 6.25	> 70	11,312.7 (8286.5-15,139.3)	−18.59	0.16
England SDI (0.79–0.85)	Age (Years)				Age (Years)			
	A.S	3944.3 (3130.5-4983.2)	−26.17	17.71	A.S	5276 (4205.2-6697)	− 23.35	17.65
	15–49	4729.9 (3392.7-6424.5)	−34.8	30.85	15–49	6484.2 (4706.8-8814.4)	−30.52	30.69
	50–69	7365.6 (4794.8-11,048.3)	−18.21	5.44	50–69	9474.7 (6180.2-14,146.2)	−20.14	7.33
	≥70	7351 (4920.9-11,069.5)	−17.08	−3.25	> 70	8844.8 (6019.7-12,806.3)	−23.79	−6.94

Global, Brazilian, American and England prevalences and YLDs by sex, age-standardized (A.S) and age range rates in 2019, and percentage change (▲v%) from 2000 to 2010 and from 2010 to 2019. Social-Demographic Index (SDI) from 2000 and from 2019

The global estimates show that, worldwide, aging was associated with a significant increase in neck pain prevalence. In 2019, there was a statistical difference in the global prevalence of neck pain for both sexes in the age group of 15 to 49 years, where the estimates were 2770.4 (95% IU 2027.9 – 3851.4) per 100,000 population; in the age group of 50 to 69 years, where the estimates were 6064.2 (95% IU 4070.1 – 9132.7) per 100,000 population; and in the age group of 70 years and older, with prevalence estimates of 5971.6 (95% IU 4080 – 8621.7) per 100,000 population. However, when assessing the data in the countries evaluated, with the exception of the USA, there was only a tendency for this increase in prevalence with aging (Table 3).

Neck pain was the ninth clinical condition with the highest disability burden among the 369 diseases assessed by the GBD study in 2019, with a global age-standardized rate of 267.4 YLD (95% IU 175, 5–383.5) per 100,000 population. In Brazil, in 2019, it represented the sixteenth clinical condition with the greatest impact on the population health, with a standardized rate for YLD of 221.7 (95% IU 145.4–322.6) per 100,000 population. Contrary to the prevalence estimates, there was no statistical difference between the YLD values comparing Brazil, Mexico, the USA and England in 2010 and 2019. According to the estimates presented in Table 2, the USA and England had, with the exception of the year 2000, a trend of higher YLD rates, while Brazil and Mexico tended to lower values that were closer to the global numbers.

The age-standardized YLD rates were very similar among the Brazilian states. The state of São Paulo showed a trend to a higher burden of disability, with 328.5 YLD (95% IU 216.3–476.6) per 100,000 population in 2019, but with coincident confidence intervals.

## Discussion

This study shows a similar prevalence of neck pain among the global and countries with lower SDI (Brazil and Mexico), in contrast to higher estimates in countries with higher SDI (England and USA). Although generalization is not possible, other studies also showed a higher prevalence and burden of neck pain among countries with higher SDI [5, 23]. On the other hand, population aging is increasing exponentially in countries with lower SDI, under conditions of low access to diagnosis and treatment of chronic-degenerative diseases [25]. Barriers to accessing health care and, consequently, receiving a diagnosis, may explain the underestimation of data in these low-income countries [26]. In 2013, a first Brazilian survey on spinal pain indicated an increase from 18.5% (95%UI 17,8-19,1) (2013) to 21.6% (95%UI 21–22.1) (2019), with subnational variation in 2019 from (13 95%UI 1.6–15.7) in the Federal District, to 26% (95%UI 23.2–28.8) in Bahia. This survey indicates spinal pain affects about 34.3 million Brazilians, but data did not distinguish the specific local of spinal pain [27].

The annual change in prevalence and YLDs estimates of neck pain in Brazil and in all of its federative units did not follow the same patterns presented by the other countries. While global and Mexican estimates were stable over the period evaluated, Brazilian prevalence estimates increased between 2000 and 2010 and then decreased between 2010 and 2019. The opposite pattern was presented by countries with a higher SDI such as the USA and England. These differences can be mainly attributable to the methodologies and data sources used in the epidemiological studies performed in each location.

The stability and homogeneity observed in the global prevalence estimates, as well as for Brazil, and Mexico, suggest the absence of population-based prevalence studies. Likewise, most of the Brazilian federative units presented similar prevalence estimates and variations between the years evaluated, with the notable exception of São Paulo. This result suggests a paucity of data source and scientific data in Brazil in this period, since among the three studies carried out in Brazil [7, 15, 24] two were carried out in São Paulo [7, 24].

According to the results, the prevalence of cervical pain in Brazil ranges from 20.3% (95% CI 18–22.7%) to 22% (95% CI 19.3–25%). The third study, carried out in the state of Rio de Janeiro, covered only among young adults, ages ranging from 18 to 21 years old, and presents a prevalence of 36% (95% CI 28.4–44.3%) [15]. The Brazilian national health surveys do not address specifically the neck pain. Methodological differences between theses studies and GBD estimates do not allow comparison of results.

Previous studies pointed out the higher prevalence rate of neck pain among women [9, 28, 29], and their lower tendency to recover from such pain [30], different from data of more recent studies [7, 23]. A higher prevalence of chronic spinal pain among women was also reported in Brazil [31]. GBD estimates in 2019 showed higher prevalence only among USA women, while the other studied countries did not present the same trend. These differences may represent different exposure to work-related risk factors and sedentary life-style as well as different access to diagnosis in these countries.

The higher prevalence of neck pain among middle-aged adults and elderly shows the importance of age as a risk-factor to of aging musculoskeletal diseases [9, 31–35]. Studies have only shown a significant peak of point prevalence in the middle age [5, 23, 30, 34]. Younger men and women have a higher prevalence of neck pain, but recover more often from it than older individuals. The overall rates of recurrence and chronicity of neck pain are around 30 to 50% [8, 36]. This chronicity, added to the fact that it affects both sexes and begins at a younger age, make the burden of neck pain to be classified among the highest YLDs in Brazil and in the world [37].

Despite the prevalence of neck pain being statistically higher in the higher-income countries evaluated, the USA and England, this difference was not significant between the YLDs when compared to the estimates of Brazil.. This fact suggests that neck pain affects a younger population in Brazil, the long duration of the disease make the YDL similar to countries with a higher prevalence.

Despite the prevalence of neck pain is statistically higher in the higher SDI countries evaluated, this difference was not significant between the YLDs when compared to the estimates of Brazil. Musculoskeletal conditions are neglected in most national health surveys: among 170 national health surveys from different countries, only 37 cited cervical pain [38]. Besides, the different definitions of neck pain prevent the comparison [38]. The higher rates presented in the US are probably related to the inclusion of neck pain investigation in annual surveys since 2013, the availability of regular data providing more reliable information.

Low income is a risk factor for chronic spinal pain and is associated with a worse prognosis for chronic neck pain [7, 31]. More recent studies have revealed a higher prevalence of chronic neck pain and a notable association with people with low education [39, 40]. Thus, since the burden of the disease depends on the sum of the prevalence of the different spectra of the disease, which in the case of neck pain encompasses severity and chronicity, the fact that lower SDI countries have a YLD similar to that of higher SDI could be justified by a higher prevalence of chronic and severe neck pain in these locations.

The GBD Project systematically updates global and national estimates of the burden of several diseases, aiming to encourage detailed analysis for decision making at a local level. Regarding the scientific production using GBD estimates, the GBD Brazil Network has done several analyses that have resulted in the publication of two capstone papers and two journal supplements on the burden of several diseases [17]. This study presents the current Brazilian status of the burden of neck pain with the best available data. Considering Brazilian subnational diversity, obtaining reliable estimates is challenging. Our results emphasize the importance of the research on musculoskeletal diseases in the country, especially in the current context of economic and political crisis, with funding restriction [41].

This study presents as a strength the standardized methodology that enables comparisons between locations and times. On the other hand, it does not distinguish between the risk factors associated with the onset and recurrence of cervical pain, nor does it analyze the risk factors common to cervical pain, such as exposure to high-demand work, smoking history, psychological disorders, low educational level and a past history of cervical pain [42, 43], since these were not addressed by the GBD study.

Given the trend for population aging, greater investments in public health policies are necessary to avoid individual and financial losses. The results of this study can contribute to the understanding of the importance of neck pain and help improve the prevention and control of risk factors for chronic degenerative diseases programs. In addition, it highlights the importance of diagnosis, early treatment and adequate rehabilitation, which tend to reduce the disability generated by neck pain.

## Data Availability

Data we used in this article are publicly available online (http://ghdx.healthdata.org/gbd-results-tool).

## Abbreviations

A.S: Age-Standardized
DALY: Disability adjusted life years
DW: Disability weight
GBD: Global Burden of Diseases
ICD-10: International Classification of Diseases, tenth edition
IHME: Institute for Health Metrics and Evaluation
P: Prevalence
SDI: Socio-Demographic Index
UFMG: Federal University of Minas Gerais
UI: Uncertainty intervals
USA: United States of America
YLD: Years lived with disability
YLD_NP_: YLD due to neck pain
YLL: Years of life lost due to premature mortality
